# Continuous Glucose Monitoring–Guided Insulin Infusion in Critically Ill Patients Promotes Safety, Improves Time Efficiency, and Enhances Provider Satisfaction

**DOI:** 10.1016/j.eprac.2025.05.751

**Published:** 2025-06-23

**Authors:** Erin R. Giovannetti, Rachael O. Lee, Robert L. Thomas, Tamar Wolinsky, Adrianne V. Talbot, Rabia S. Ali, Tricia Santos Cavaiola, Kristen Kulasa, Schafer C. Boeder

**Affiliations:** 1 Inpatient Glycemic Control, UC San Diego Health, La Jolla, California; 2 Patient Care Services, University of California, Davis, California; 3 Division of Endocrinology and Metabolism, Department of Medicine, University of California, San Diego, La Jolla, California

**Keywords:** real-time continuous glucose monitoring (rtCGM), intensive care unit (ICU), mean absolute relative difference (MARD), surveillance error grid (SEG), clinician turnaround time (TAT)

## Abstract

**Objective:**

Evaluate the integration of real-time CGM (rtCGM) into an insulin infusion computer calculator (IICC) to improve glycemic control, time efficiency, safety, and clinician workflow in the intensive care unit (ICU).

**Methods:**

A retrospective analysis was conducted on 35 critically ill adult patients requiring insulin infusion in the surgical and medical ICUs. Dexcom G7 rtCGM values were integrated into an institution-developed IICC using an ongoing validation protocol, allowing for nonadjunctive CGM use. The accuracy of rtCGM was assessed by comparing matched CGM and point-of-care (POC) glucose values using mean absolute relative difference (MARD), surveillance error grid, and Parkes Error Grid analyses. CGM time-in-range metrics, clinician turnaround time for glucose monitoring, and nurse satisfaction were also evaluated.

**Results:**

A total of 1291 matched glucose pairs were analyzed. The rtCGM system demonstrated a MARD of 12.5%, with 99.6% of the values falling within clinically acceptable error zones (A+B) on the Parkes Error Grid. Patients in the rtCGM-IICC protocol had mean glucose 141.9 mg/dL, with mean time in range (70–180 mg/dL) 82.8%, time above range (> 180 mg/dL) 14.5%, and time below range (< 70 mg/dL) 0.5%. Clinician time efficiency improved significantly, with POC testing requiring a mean turnaround time of nearly 5 minutes compared to 3-second CGM retrieval. All surveyed nurses (*n* = 20) reported rtCGM increased efficiency and improved safety and preferred rtCGM with POC over POC testing alone.

**Conclusion:**

Integrating rtCGM with an IICC protocol in the ICU enhances glycemic control, improves workflow efficiency, and reduces clinician workload while maintaining high accuracy.

## Introduction

Inpatient glucose control during critical illness has been a subject of intense investigation for decades. While uncontrolled hyperglycemia is clearly harmful, efforts to implement stricter inpatient glucose control have met with mixed results, including increased mortality due to hypoglycemia.^1,2^ At present, glycemic monitoring for patients on titratable insulin infusion is invasive and labor-intensive, requiring hourly point-of-care (POC) glucose monitoring. Continuous glucose monitoring (CGM) offers a promising alternative for inpatient glycemic management. Unlike intermittent POC glucose testing, CGM provides real-time continuous data and alerts, enabling clinicians to monitor glycemic trends and patterns more effectively. While CGM is a well-validated tool for outpatient use, where it has been shown to improve glucose regulation and reduce glycemic variability,^3^ its role in the inpatient setting remains under investigation.

Recognizing the potential of CGM technology in the inpatient setting, the U.S. Food and Drug Administration granted Dexcom’s CGM system a Breakthrough Device Designation to facilitate the evaluation of its performance within hospital environments.^4^ This designation underscores the growing evidence supporting CGM’s ability to improve glycemic control in inpatient settings. Previous studies have shown that inpatient CGM use improves staff satisfaction while maintaining a high standard of patient care^5,6^ and allows health care teams to effectively manage the dynamic challenges of glycemic control in hospitalized patients, promoting safe and efficient glycemic management.^7–10^

In the intensive care unit (ICU), maintaining stable blood glucose levels is especially challenging due to physiological stress, heightened inflammatory responses, disrupted nutritional intake, fluctuating organ function, and the impact of various medications such as corticosteroids, vasopressors, and immunosuppressants. These factors complicate glucose regulation, often leading to hyperglycemia or hypoglycemia, both of which are associated with poorer patient outcomes. CGM helps address these complexities by providing customizable alerts for critical deviations (hypo- and hyperglycemic events) and enabling clinicians to implement timely and actionable interventions that enhance patient safety and outcomes.

As the clinical community continues to explore CGM’s role in critical care, the evidence suggests a paradigm shift in glycemic management, with significant implications for patient safety and hospital efficiency. This study aims to demonstrate that, in addition to improving clinical outcomes, CGM enhances clinician workflow efficiency and increases staff satisfaction by minimizing workload—without increasing hypoglycemia risk.

## Materials and Methods

### Study Design and Participants

This retrospective study evaluated the use of real-time CGM (rtCGM) for glycemic management in critically ill patients requiring insulin infusion therapy in the ICU. The study was granted an exemption by our Institutional Review Board under Code of Federal Regulations category 45 CFR 46.104(d), allowing retrospective analysis using deidentified demographic data and anonymized blood glucose and CGM values extracted from electronic health records (EHRs). Eligible patients met the following criteria: age > 18 years, admitted to the surgical ICU or medical ICU, and required insulin infusion therapy for glycemic management. The inclusion criteria encompassed all critically ill patients including those with hemodynamic instability, peripheral edema, vasopressor support, and those receiving extracorporeal membrane oxygenation (ECMO) or continuous renal replacement therapy (CRRT) to assess CGM accuracy and reliability in a high-acuity population. The only exclusion criterion was ICU admission without concurrent insulin infusion therapy.

### Insulin Infusion Computer Calculator

Our institution previously developed an insulin infusion computer calculator (IICC) using a dynamic, coefficient-based algorithm integrated directly into the electronic medication administration record of our EHR to generate safe and effective dosing for continuously administered intravenous regular insulin.^11^ The IICC adjusts insulin infusion rates based on the current glucose value, the rate of change from the last entry, and changes in nutrition. The IICC is utilized throughout our health system for patients with various conditions including critical care hyperglycemia, diabetic ketoacidosis, hyperosmolar hyperglycemic state (HHS), and hyperglycemia induced by steroids, tube feeds, or parenteral nutrition.

### Continuous Glucose Monitor Utilization Protocol

Continuous glucose monitoring was implemented in the ICU through a clinical protocol that integrated CGM values into the IICC using a validation system, allowing nonadjunctive CGM values to guide insulin infusion rates. The protocol was approved by our institution’s Aligning and Coordinating Quality Improvement, Research, and Evaluation Committee. The hybrid POC glucose testing plus CGM model utilized in this study was originally pioneered by Faulds et al^10^ and has since been adopted in subsequent research. Our protocol used the Dexcom G7 CGM device (Dexcom Inc). Critical care leadership, critical care clinical care specialist, and inpatient certified diabetes care and education specialists assisted in training staff on the CGM protocol. The sensor was placed on the upper posterior arm by a member of the inpatient glycemic control team (critical care nurse or certified nurse practitioner) and required a 30-minute warm-up period before displaying CGM glucose values. An iPhone, used as the CGM receiver, was positioned within 20 feet of the patient to ensure Bluetooth connectivity.

**Fig. 1.**
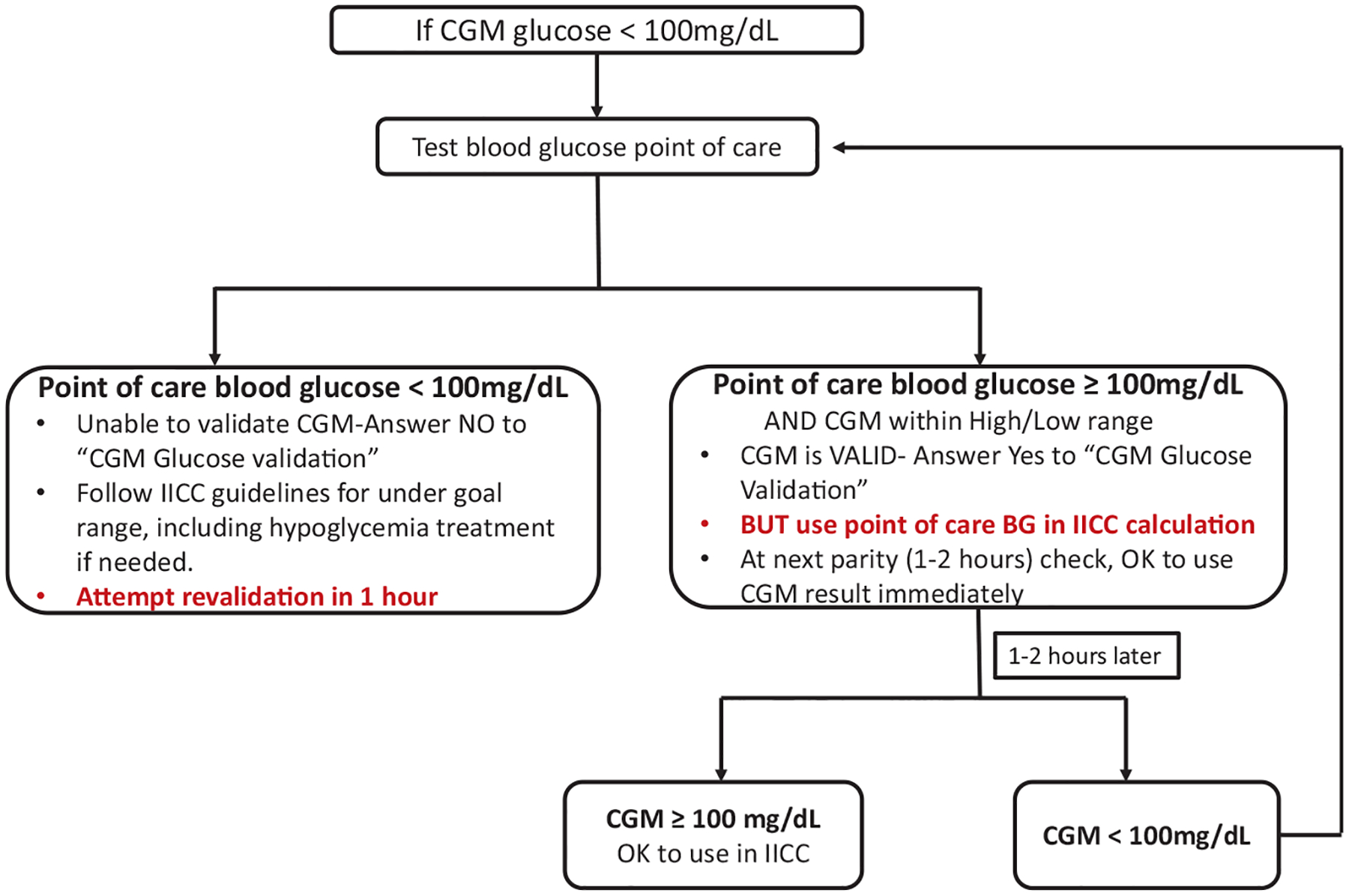
Validation algorithm for use of CGM with IICC. Verification of concordant CGM and POC glucose values > 100 mg/dL allows subsequent insulin titration using CGM readings. CGM glucose < 100 mg/dL triggers new POC glucose and revalidation. *CGM* = continuous glucose monitoring; *IICC* = insulin infusion computer calculator.

Continuous glucose monitor validation was done in 2 stages: initial validation with each new sensor and ongoing validation throughout the sensor use while the patient remained on the IICC. For initial validation, 2 consecutive POC glucose confirmations, taken at least 1 hour apart, were required before rtCGM could be used nonadjunctively. Validation thresholds were calculated within the EHR, requiring CGM values to be within ± 20% of the POC measurement (capillary, venous, or arterial blood) obtained using the Accu-Chek Inform II blood glucose meter (Roche Diabetes Care GmbH). Continuous glucose monitor values were considered validated if they met this threshold and the corresponding POC glucose was >100 mg/dL. For continued nonadjunctive use, POC glucose was checked every 6 hours to maintain validation. If validation failed—due to CGM values falling outside the ± 20% threshold, POC glucose < 100 mg/dL, or CGM values not displaying—a single successful repeat validation was required (Fig. 1).

To ensure actionable alarms, the CGM low alert was set at 100 mg/dL. Under our academic institution’s IICC protocol, the target glucose range in the ICU was 100 to 150 mg/dL. If CGM values fell below 100 mg/dL, POC glucose was tested. If the POC results confirmed glucose < 100 mg/dL, insulin infusion was paused, and POC monitoring increased to every 15 minutes for 2 consecutive checks. If glucose levels rose above 100 mg/dL, hourly monitoring resumed. If CGM values remained within ±20% of POC readings, rtCGM data continued to guide insulin infusion rates. Patients remained on the CGM IICC protocol as long as they required insulin infusion in the ICU.

### Data Collection and Statistical Analysis

Time-matched CGM and POC glucose values were extracted from the EHR for CGM accuracy analysis. Key performance measures included the mean absolute relative difference (MARD), standard deviation relative difference (SDRD), relative bias, and the upper and lower 95% limits of agreement. The MARD was calculated across all matched pairs of POC and G7 values. To assess accuracy and clinical relevance, we performed surveillance analysis, Parkes error grid analysis, and Clarke error grid analysis,^12–14^ which visually stratify data across 5 clinical risk levels. Additionally, a modified Bland-Altman plot was used to illustrate the bias between the POC and CGM values.^15^

Patient demographic data—including age, sex, race or ethnicity, body mass index, diabetes history, clinical features, and illness severity—were also collected and analyzed in aggregate. CGM metrics, including mean glucose, time in range (70–180 mg/dL), time above range (> 180 mg/dL), time below range (< 70 mg/dL), and time very low (< 54 mg/dL), and coefficient of variation were obtained from the Dexcom Clarity database. The CGM metrics were collected from initiation to the discontinuation of the CGM-IICC protocol, reflecting the entire duration of use rather than daily values.

To assess clinician time efficiency, we conducted an observational analysis comparing the turnaround time (TAT) for obtaining POC glucose values versus retrieving CGM glucose values. A research team member used a stopwatch to measure the time required for each task, starting at initiation and stopping upon completion. Additionally, we assessed clinicians’ perception of time efficiency, safety, accuracy, and overall satisfaction using a 7-item survey administered electronically using Qualtrics XM platform. Responses were recorded on a 5-point Likert scale.

Statistical analysis was conducted using Microsoft Excel (Microsoft Office 2024, Microsoft Corporation), Prism 10 statistical software version 10.4.1 (GraphPad Inc), and online tools provided by the Diabetes Technology Society.^16^

## Results

### Demographics

Our study analyzed data from 35 adult ICU patients requiring insulin infusions (Table 1). The median CGM wear time was 3 days (IQR: 1–9 days). The cohort had a median age of 62 years (range: 23–84 years) and a median body mass index of 27.9 kg/m^2^. Males comprised 60% of the group, and 57.1% had type 2 diabetes. The median hemoglobin A1c was 6.6% (49 mmol/mol) with a range of 4.5% to 15.1% (26–142 mmol/mol).

A substantial proportion of the cohort required advanced supportive therapies. Of the 35 patients, 28 required mechanical ventilation, 27 required titratable intravenous vasopressors, 25 received glucocorticoids, and 25 relied on parental nutrition or tube feeding. Additionally, 10 patients underwent CRRT and 5 required ECMO. The median Sequential Organ Failure Assessment score was 10, indicating significant organ dysfunction with an associated risk of in-hospital mortality in the range of 40% to 50% or higher.^17^ The actual in-hospital mortality rate in our study was 22.9%, reflecting the severity of illness within the cohort.

### Accuracy and Device Performance

**Fig. 2.**
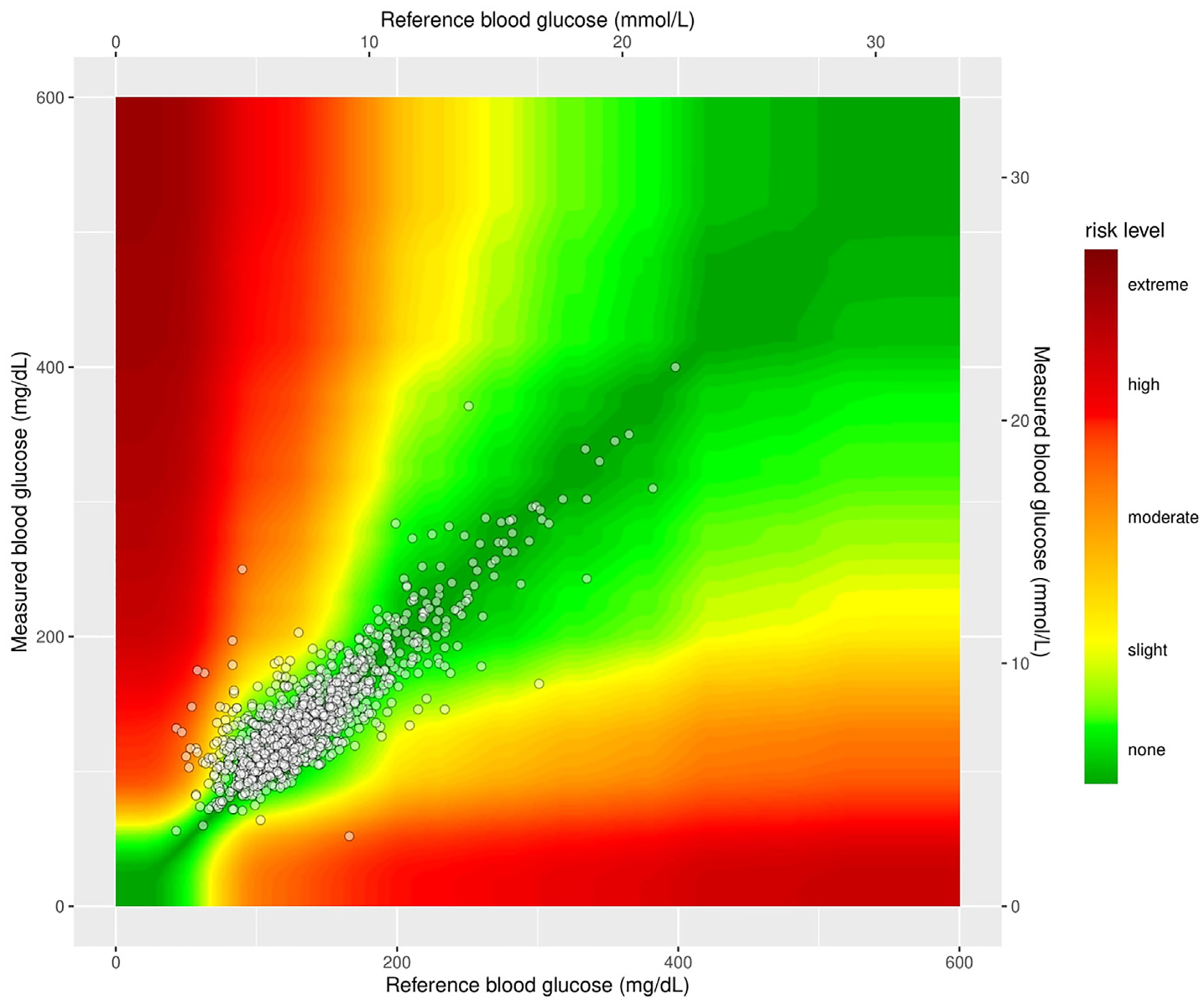
Surveillance error grid of CGM (Y) versus POC (X) glucose in critically ill patients; 99.6% of CGM values fall within acceptable error zones A (clinically accurate) + B (minor inaccuracy with no clinical impact) compared with typical POC glucose monitoring. Calculations per Blood Glucose Monitoring System Surveillance Program, https://www.diabetestechnology.org/seg/, last accessed February 5, 2025. *CGM* = continuous glucose monitoring; *POC* = point of care.

**Fig. 3.**
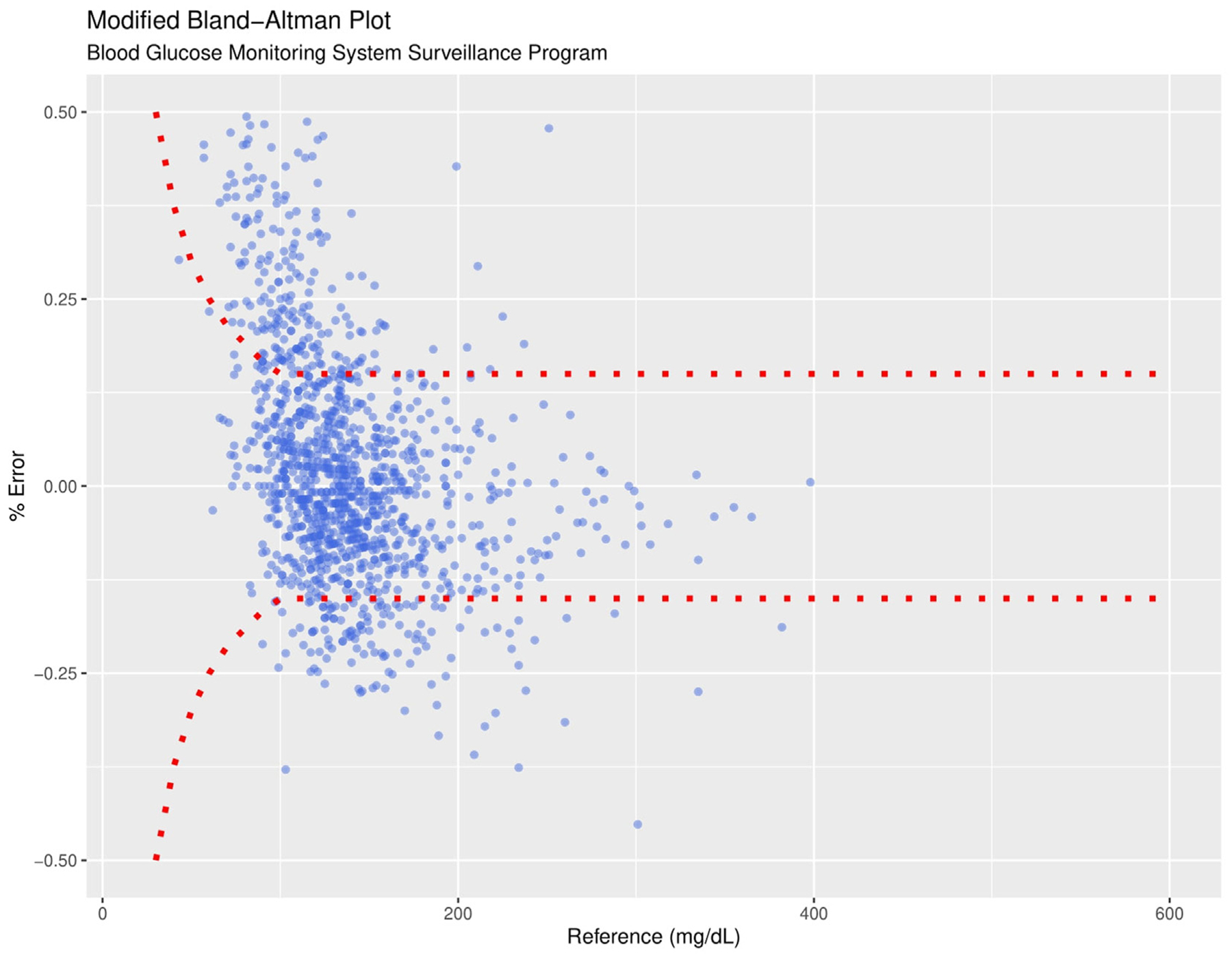
Modified Bland-Altman plot of paired glucose values from CGM and POC reference measurements. Red borders represent a 15 mg/dL (if < 100 mg/dL) or 15% (if > 100 mg/dL) difference limit from POC glucose values. *CGM* = continuous glucose monitoring; *POC* = point of care.

To assess CGM accuracy, we compared 1291 rtCGM values with matched POC glucose measurements. The MARD for rtCGM was 12.5% and the SDRD was 17.8%. The relative bias was 1.5%, with 95% limits of agreement ranging from 36.3% to 33.3%. To assess clinical accuracy, we utilized the surveillance error grid, which categorizes glucose readings based on potential clinical risk.^13^ In our analysis, 99.1% of rtCGM values fell within zones A and B, with 0.9% in zone C and 0.1% in zone D (Fig. 2). Similarly, using the Parkes error grid, 99.6% of values were in zone A (clinically accurate) or zone B (minor inaccuracy with no clinical impact), while 0.4% fell in zone C (likely to affect clinical outcome) and 0.1% in zone D (significant clinical risk) (Table 2). These findings demonstrate that the rtCGM system meets the clinical accuracy standards set by the International Organization Standardization, which require 99% of measurements to fall within zones A and B of the Parkes error grid (also known as the consensus error grid). The 15 mg/dL (for < 100 mg/dL) or 15% (for > 100 mg/dL) difference limit between rtCGM and POC glucose values is shown (Fig. 3). Notably, one trauma patient with multiple organ injuries, including 19% total body surface area burns, remained in the surgical ICU for 66 days while receiving ECMO, mechanical ventilation, vasopressor support, and enteral tube feeding. The CGM remained accurate throughout the hospitalization, with a MARD of 13.2% during the first 2 months and a lower MARD of 7.5% in the last 2 weeks. This case highlights the value of CGM for longitudinal ICU care and reinforces the importance of early validation with POC glucose measurements.

### CGM Metrics

In our cohort using rtCGM with the IICC, the mean CGM glucose was 141.9 mg/dL. On average, patients spent 82.8% of the time within target range for glucose (70–180 mg/dL), 14.5% above range (>180 mg/dL), 0.5% below range (< 70 mg/dL), and 0.1% at very low levels (< 54 mg/dL). Notably, the average coefficient of variation was 25.2% indicating stable glucose levels and well within the target threshold of less than 36% for inpatient CGM studies.^18^ In an analysis of 1291 matched pairs of rtCGM and POC glucose measurements, only 0.2% (3 values) of POC readings were below 70 mg/dL, with none falling below 54 mg/dL. Notably, one patient presented with severe diabetic ketoacidosis and HHS. For this individual, the IICC glucose target was adjusted to 200 to 250 mg/dL for 24 hours, in accordance with standard HHS management guidelines. This adjustment accounted for the majority (10.8% of the total 14.5%) mean time above range across all patients.

### Observational Turnaround Time and Survey

We assessed the TAT for bedside POC glucose testing using the Accu-Chek Inform II meter compared to rtCGM. This evaluation included all steps involved in obtaining a glucose measurement. For POC testing, this included gathering supplies (for capillary, venous, or arterial testing), accessing the operator screen, scanning test strips and patient identification, and performing the test. Factors affecting TAT included quality control checks, repeat sampling due to insufficient blood volume, and interruptions from critical care interventions such as responding to CRRT, ventilator, or ECMO alerts. The mean TAT for POC testing was 4 minutes and 59 seconds (median: 4 minutes and 18 seconds), consistent with prior ICU findings of 5 to 8.5 minutes^19^ and hospital-based studies of under 5 minutes.^20^

To assess clinician perception of time efficiency, perceived safety, accuracy, and satisfaction, a survey was administered. All 20 participating nurses self-reported that checking rtCGM glucose values took under a minute. For POC testing, one nurse self-reported TAT < 1 minute, 11 reported 1 to 3 minutes, and 8 reported 4 to 6 minutes, aligning with observed TAT data. All nurses agreed that rtCGM reduced time spent on glucose monitoring, and 100% preferred a combination of rtCGM and POC glucose over POC testing alone. Importantly, all nurses agreed that CGM improved patient safety and expressed satisfaction with using rtCGM for insulin infusion monitoring.

## Discussion

The integration of rtCGM into critical care settings, particularly for ICU patients on insulin infusions, offers significant benefits. Our study demonstrates that rtCGM, when used in real-world settings with ongoing clinician validation and an IICC, provides high accuracy glucose measurements and maintains patient safety with low hypoglycemia rates. Specifically, just 0.2% of matched CGM and POC glucose values were <70 mg/dL, and we observed a 0.5% time below 70 mg/dL and 0.1% time below 54 mg/dL based on CGM metrics. Severe hypoglycemia remains a major concern in intensive insulin therapy due to its association with increased mortality and adverse neurological outcomes. Traditional blood glucose monitoring, which relies on intermittent POC measurements, may miss critical glucose fluctuations, leading to delayed detection of both hypo- and hyperglycemia. By providing continuous glucose data along with alarm capabilities, rtCGM enables earlier intervention and a more proactive approach to glucose management. Our protocol, in which a POC test was performed when glucose levels fell below 100 mg/dL, ensured timely confirmation and intervention, and further enhanced patient safety. Our findings align with previous studies supporting the safe use of rtCGM in critically ill patients, including those with shock, edema, and ECMO.^9,21^

**Fig. 4.**
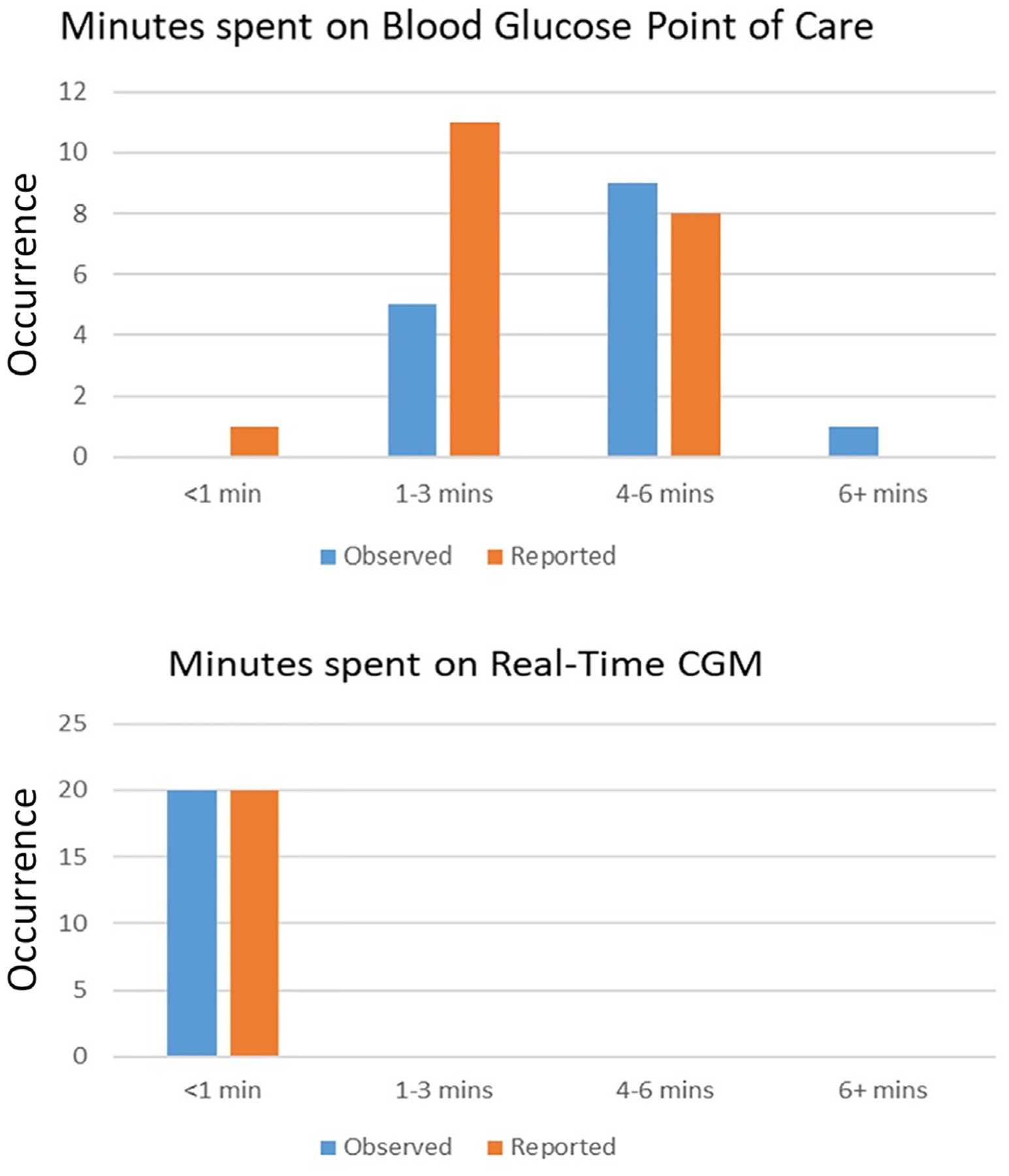
Observed and reported TAT for POC and CGM glucose checks. TAT is significantly reduced with CGM monitoring compared to typical POC glucose checks. All CGM glucose checks were < 1 minute (average 3 seconds), while POC glucose checks took an average of 5 minutes. *CGM* = continuous glucose monitoring; *POC* = point of care; *TAT* = turnaround time.

The low MARD of 12.5% between CGM and POC measurements, with 99.6% within zone A and B of the consensus error grid, further underscores the reliability of rtCGM in the ICU settings. While other studies have reported MARD values of 13.2%^21^ and 13.9%^9^ using a hybrid POC-CGM protocol for titration of an insulin infusion, our study is the first to show improved accuracy using the Dexcom G7 sensor. Another key advantage of rtCGM is its significantly faster TAT compared to POC glucose testing. While POC testing takes an average of nearly 5 minutes, rtCGM provides glucose readings in 3 seconds, offering substantial time savings (Fig. 4). Over a 12-hour nursing shift, this could save an estimated 50 minutes, allowing more time for other critical patient care tasks. The reduction in manual glucose testing not only enhances efficiency but also reduces the burden on ICU staff, supporting overall workflow improvements.

In our study, critical care nurses reported high satisfaction with rtCGM implementation, citing reduced workload, improved efficiency, and perceived patient safety as key benefits. Additionally, rtCGM alarm capabilities and glucose trend arrows facilitated earlier recognition of impending hypoglycemia, allowing clinicians to intervene before critical glucose thresholds were reached. Given that ICU clinical decisions rely heavily on continuous physiologic monitoring, rtCGM has the potential to function as a vital sign itself, providing continuous glucose data rather than intermittent glycemic snapshots. This more proactive approach to glycemic monitoring and management could not only improve glycemic outcomes but may also help optimize resource utilization by reducing the frequency of POC testing and manual insulin adjustments. As a result, CGM-guided protocols have the potential to lower overall health care costs while enhancing patient safety.

We acknowledge the small sample size (*N* = 35) and single-site, retrospective design limits the generalizability of our findings. However, we were encouraged by the diversity of our study population, including 45% non-White participants. Despite this strength, limitations in statistical power and demographic breadth should be considered when interpreting the results. While our study demonstrates potential benefits of CGM, there are areas that warrant further exploration. First, future studies should explore the optimal calibration strategies for CGM use in critically ill populations, as recent studies suggest that calibration frequency may impact MARD and SDRD in highly dynamic glucose environments.^22,23^ Additionally, further research is needed to refine hospital-wide CGM-driven protocols, particularly in integrating rtCGM into the EHR for streamlined workflow implementation. Studies by Finn et al^24^ and Lee et al^5^ have demonstrated the feasibility of CGM policies following hospital-wide EHR integration across acuity levels. Investigating these factors will be essential to refining CGM as a clinical parameter and maximizing its impact on patient outcomes.

In conclusion, integrating rtCGM into the management of ICU patients on insulin infusions provides a promising strategy for enhancing patient safety, improving clinical outcomes, and reducing clinician workload. The combination of real-time glucose data and algorithm-driven insulin dosing offers an effective, efficient approach to managing glycemic variability in critically ill patients. By mitigating hypoglycemia risk, improving glycemic stability, and reducing the burden of frequent glucose monitoring, rtCGM has the potential to transform ICU glycemic management. As hospitals increasingly adopt digital health innovations, future research should focus on validating CGM-driven protocols, training clinician teams on CGM workflows, investigating rtCGM as a safety-net for prompting clinically significant unscheduled POC checks, and assessing the longitudinal impact of CGM implementation on morbidity and mortality in critically ill populations.

## Figures and Tables

**Table 1:** Patient Characteristics

Characteristics	n	% or range
Age (median)	62	23–84
Biologic sex		
Male	21	60%
Female	14	40%
Race or ethnicity		
Hispanic or Latino	11	31.40%
White, non-Hispanic	9	25.70%
Black or African American	1	2.90%
Asian	4	11.40%
Unknown or not specified	10	28.60%
BMI (median)	29.3	17.79–41.55
>30	14	40.00%
18–29.9	20	57.10%
<18	1	3%
Diabetes history		
None or unknown	7	20.00%
Pre-diabetes	3	8.60%
DM2	20	57.10%
DM1/MODY	2	5.70%
Pancreatic DM	3	8.60%
A1C on admission, % (median)	6.6	4.5%−15.1%
A1C on admission, mmol/mol (median)	49	26–142
Interventions and clinical status		
Inpatient mortality	8	22.90%
Mechanical ventilation	28	80.00%
Vasopressors	27	77.10%
Glucocorticoids	25	71.40%
Parental or enteral nutrition	25	71.40%
SOFA (median)	10	0–18
ECMO	5	14.30%
CRRT	10	28.60%

Abbreviations: BMI = body mass index; CRRT = continuous renal replacement therapy; ECMO = extracorporeal membrane oxygenation; MODY, maturity onset diabetes of the young; SOFA = Sequential Organ Failure Assessment.

**Table 2:** DTS, Surveillance, Parkes, and Clarke Error Grid Risk Zone Comparisons of Paired rtCGM and POC Glucose Values

Risk zone	DTS EG count	DTS EG frequency	SEG count	SEG frequency	Parkes count	Parkes frequency	Clarke count	Clarke frequency
A: No risk	1044	80.9%	1110	86.0%	1053	81.6%	1045	80.9%
B: Mild risk	231	17.9%	169	13.1%	232	18.0%	242	18.7%
C: Moderate risk	15	1.2%	11	0.9%	5	0.4%	1	1.0%
D: High risk	1	1.0%	1	0.1%	1	0.1%	3	2.0%
E: Extreme risk	0	0.0%	0	0.0%	0	0.0%	0	0.0%
Not included	0	0.0%	0	0.0%	0	0.0%	0	0.0%

Abbreviations: DTS = Diabetes Technology Society; POC = point of care; real-time continuous glucose monitoring; SEG = surveillance error grid.

## Highlights

- Real-time continuous glucose monitoring (rtCGM) using Dexcom G7 sensors achieved a low mean absolute relative difference of 12.5% compared with point-of-care measurements among critically ill patients requiring advanced supportive therapies
- Intensive care unit rtCGM implementation reduced time spent on glucose measurement by approximately 50 minutes per nursing shift for each patient
- Participating providers were highly satisfied with rtCGM for insulin infusion monitoring and agreed that rtCGM alerts and trends improve efficiency and patient safety

## Clinical Relevance

Modern real-time continuous glucose monitors and insulin infusion computer calculators can achieve high accuracy glucose measurements, high time in target range, and low hypoglycemia rates in real-world intensive care unit settings. These systems can also reduce clinician workload and provide early warnings to help prevent dangerous glucose excursions.

## Abbreviations:

CGM: continuous glucose monitoring
CRRT: continuous renal replacement therapy
ECMO: extracorporeal membrane oxygenation
EHR: electronic health record
HHS: hyperosmolar hyperglycemic state
ICU: intensive care unit
IICC: insulin infusion computer calculator
MARD: mean absolute relative difference
POC: point-of-care
rtCGM: real-time CGM
SDRD: standard deviation relative difference
TAT: turnaround time
